# DXA-based Fat Mass With Risk of Worsening Insulin Resistance in Adolescents: A 9-Year Temporal and Mediation Study

**DOI:** 10.1210/clinem/dgae004

**Published:** 2024-01-04

**Authors:** Andrew O Agbaje, Christoph Saner, Jie Zhang, Mélanie Henderson, Tomi-Pekka Tuomainen

**Affiliations:** Institute of Public Health and Clinical Nutrition, School of Medicine, Faculty of Health Sciences, University of Eastern Finland, Kuopio, 70211, Finland; Children’s Health and Exercise Research Centre, Department of Public Health and Sports Sciences, Faculty of Health and Life Sciences, University of Exeter, Exeter, EX1 2LU, UK; Division of Pediatric Endocrinology, Diabetology and Metabolism, Department of Pediatrics, Inselspital, Bern University Hospital, University of Bern, Bern, CH-3010, Switzerland; Department of Biomedical Research, University of Bern, Bern, CH-3010, Switzerland; Department of Public Health, Aarhus University, DK-8000 Aarhus, Denmark; School of Public Health, Faculty of Medicine, Université de Montréal, Montréal, Quebec, H3T 1C5, Canada; Department of Pediatrics, Faculty of Medicine, Université de Montréal, Montréal, Quebec, H3T 1C5, Canada; Research Center of Centre Hospitalier Universitaire Sainte-Justine, Montréal, Quebec, H3T 1C5, Canada; Institute of Public Health and Clinical Nutrition, School of Medicine, Faculty of Health Sciences, University of Eastern Finland, Kuopio, 70211, Finland

**Keywords:** obesity, pediatrics, causality, adiposity, prospective cohort study, type 2 diabetes

## Abstract

**Context:**

Surrogate measures of childhood and adolescent obesity have impaired the understanding of the relationship of body composition with insulin resistance in the young population.

**Objective:**

We aim to examine the longitudinal associations of directly measured total fat mass, trunk fat mass, and lean mass with the risk of hyperglycemia, hyperinsulinemia, and insulin resistance from ages 15 to 24 years, the mediation path through which lipids and inflammation influence insulin resistance, and whether increased fat mass temporally precede insulin resistance.

**Methods:**

We studied 3160 adolescents from the Avon Longitudinal Study of Parents and Children (ALSPAC), UK birth cohort, who had complete dual-energy x-ray absorptiometry measure and fasting blood samples at age 15 years and repeated measures at ages 17- and 24-years clinic visit. Fasting glucose greater than 6.1 mmol/L, insulin greater than 11.78 mU/L, and homeostatic model assessment for insulin resistance (HOMA-IR) greater than or equal to the 75th percentile were categorized as hyperglycemia, hyperinsulinemia, and high insulin resistance, respectively. Longitudinal associations were examined with generalized logit-mixed-effect models, while mediation and temporal path analyses were examined using structural equation models, adjusting for cardiometabolic and lifestyle factors.

**Results:**

Among 3160 participants (51% female), fat mass and lean mass increased linearly both in males and females, while glucose, insulin, and HOMA-IR had a U-shaped course from age 15 through 24 years. After full adjustment, each 1-kg cumulative increase in total fat mass (odds ratio 1.12 [95% CI, 1.11-1.13]) and trunk fat mass (1.21 [1.19-1.23]) from ages 15 through 24 years were associated with a progressively worsening risk of high insulin resistance as well as hyperglycemia and hyperinsulinemia. The association of increased total fat mass with increased insulin resistance was partly mediated by triglycerides (9% mediation). In the temporal path analysis, higher total fat mass at age 15 years was associated with higher insulin resistance at age 17 years, but not vice versa. Higher total fat mass at age 17 years was bidirectionally associated with higher insulin resistance at 24 years.

**Conclusion:**

Mid-adolescence may be an optimal time for interrupting the worsening fat mass–insulin resistance pathologic cycle and attenuating the risk of progressively worsening metabolic dysfunction before young adulthood.

The increasing rise in the prevalence of obesity in children and adolescents and the corresponding rise in the prevalence of young-onset type 2 diabetes warrants effective intervention timing aiming to attenuate this global health risk (1-3). The World Obesity Federation estimates that a quarter of a billion children and adolescents might be living with obesity by 2030 (4). It was recently reported that sedentary behavior may independently decrease insulin sensitivity in children and adolescents at risk of obesity, and increased childhood body mass index (BMI) has been associated with mid-adulthood cardiovascular morbidities and premature mortality (5-7). Several studies on the relationship between childhood and adolescent obesity have relied on surrogate measures of obesity such as BMI and waist circumference, which does not discriminate between the effect of fat mass and lean mass on metabolic alterations (3, 6-10). A direct measure of adiposity using dual-energy x-ray absorptiometry (DXA) measures of fat mass has been limited to cross-sectional studies and a few short-term longitudinal studies in small to moderate sample-sized populations (8, 10-12). Thus, large-scale, long-term prospective studies of directly measured fat mass in relation to metabolic indices are warranted to clarify the independent role of total body fat mass with respect to metabolic alteration (2, 3, 9, 10).

Moreover, it remains unknown whether increased fat mass during growth precedes metabolic alterations such as insulin resistance, or if the relationship is bidirectional in an apparently health community-based young population (8, 10). A temporal relationship has public health and clinical significance in providing evidence for the appropriate timing of intervention to limit obesity and subsequent metabolic risks. Carefully collected long-term, repeated measures of changes in exposure and outcome variables may offer evidence of a potential causal relationship between exposure and outcome when bolstered with biological plausibility. Whether increased fat mass exerts its effect on metabolic outcomes directly or via lipid, inflammation, and blood pressure pathways is not fully known and whether increased lean mass counteracts the deleterious effect of fat mass remains unclear (9, 10, 13-16). It is known that fasting glucose, insulin, and insulin resistance physiologically decrease during growth from mid-adolescence to young adulthood, and the vascular protective effect of this natural decline has been reported (17, 18). It is rather unknown if this physiologic decline has any role in attenuating increasing fat mass during postpubertal growth (3, 9, 10).

The present study (1) examined the longitudinal associations of total fat mass, trunk fat mass, lean mass, and BMI with the cumulative risk of hyperglycemia, hyperinsulinemia, and high insulin resistance at ages 15, 17, and 24 years; (2) examined the temporal and bidirectional relationship between fat mass, lean mass, and insulin resistance; and (3) assessed the extent to which the longitudinal associations of fat mass and lean mass with insulin resistance are mediated by lipid measures and inflammation using data from the Avon Longitudinal Study of Parents and Children (ALSPAC) birth cohort, England, United Kingdom.

## Materials and Methods

### Study Cohort

Data were from the ALSPAC birth cohort, which investigates factors that influence childhood development and growth. Pregnant women resident in Avon, United Kingdom, with expected dates of delivery between April 1, 1991 and December 31, 1992, were invited to take part in the study. A total of 20 248 pregnancies were identified as being eligible, and the initial number of pregnancies enrolled was 14 541. Of the initial pregnancies, there was a total of 14 676 fetuses, resulting in 14 062 live births and 13 988 children who were alive at age 1 year. When the oldest children were approximately age 7 years, an attempt was made to bolster the initial sample with eligible patient cases who had failed to join the study originally. As a result, when considering variables collected from age 7 onward (and potentially abstracted from obstetric notes) there are data available for more than the 14 541 pregnancies mentioned earlier. The number of new pregnancies not in the initial sample (known as phase I enrollment) that are currently represented in the released data and reflecting enrollment status at age 24 is 906, resulting in an additional 913 children being enrolled (456, 262, and 195 recruited during phases II, III, and IV respectively). The total sample size for analyses using any data collected after age 7 is therefore 15 447 pregnancies, resulting in 15 658 fetuses. Of these, 14 901 children were alive at age 1 year. Regular clinic visits of the children commenced at age 7 years and are still ongoing into adulthood. Study data at age 24 years were collected and managed using REDCap electronic data capture tools (19). In this study, 3160 participants who had complete directly measured body composition and fasting blood sample measures at the age 15 years clinic visits were included. Participants were followed up until age 24 years. Ethical approval for the study was obtained from the ALSPAC Ethics and Law Committee and the local research ethics committees. Informed consent for the use of data collected via questionnaires and clinics was obtained from participants following the recommendations of the ALSPAC Ethics and Law Committee at the time (20-22). Consent for biological samples has been collected in accordance with the Human Tissue Act (2004). The study website contains details of all the data that are available through a fully searchable data dictionary and variable search tool (http://www.bristol.ac.uk/alspac/researchers/our-data/).

### Exposures: Body Composition and Anthropometry

Body composition (total body fat mass, trunk fat mass, and total body lean mass) was assessed using a DXA scanner (GE Medical Systems) at 15-, 17-, and 24-year clinic visits as previously described (23-25). Repeated DXA measurements for 122 children were performed the same day, and the repeatability coefficient (twice the SD of the difference between measurement occasions) for body fat mass was 0.5 kg (13, 24, 25). Anthropometry of participants (height measured with a Harpenden wall-mounted stadiometer [Holtain Ltd]) and weight to the nearest 0.1 kg measured using a Tanita TBF-401 (Model A, Tanita Corp electronic scale) at ages 15, 17, and 24 years were assessed in line with standard protocols, and BMI was computed as weight in kilograms per height in meters squared (23, 25).

### Outcomes: Fasting Glucose, Insulin, and Insulin Resistance

Using standard protocols, fasting blood samples at ages 15, 17, and 24 years were collected, spun, and frozen at −80 °C, and a detailed assessment of fasting glucose and insulin have been described previously (23-25). Fasting insulin was measured using an ultrasensitive, automated microparticle enzyme immunoassay (Mercodia), which does not cross-react with proinsulin, and the sensitivity of the immunoassay was 0.07 mU/L (17). Participants with a fasting glucose greater than 6.1 mmol/L and insulin greater than 11.78 mU/L were categorized as at risk of hyperglycemia and hyperinsulinemia (24, 26). We calculated the homeostatic model assessment of insulin resistance (HOMA-IR) from (fasting insulin × fasting glucose/22.5) (27). HOMA-IR binary categories were grouped as greater than or equal to the 75th percentile as high and less than the 75th percentile as moderate, normal, healthy, or not high (24). The single-point insulin sensitivity estimator (SPISE) has been developed as a surrogate index for whole-body insulin sensitivity in adolescents (28). The SPISE index is computed as follows: [600×high-density lipoprotein cholesterol (HDL-c)^0.185^/(triglycerides^0.2^×BMI^1.338^)] with fasting HDL-c and triglycerides in (mg/dL), and BMI (28). To convert HDL-c to mg/dL, values in mmol/L were multiplied by 38.6, and triglycerides to mg/dL, mmol/L values were multiplied by 88.6. The Pearson correlation coefficients between the SPISE index and each of BMI, trunk fat mass, total fat mass, and lean mass were −0.90, −0.80, −0.75, and −0.37, respectively. The Pearson correlation coefficients between HOMA-IR and each of BMI, trunk fat mass, total fat mass, and lean mass were 0.37, 0.36, 0.36, and −0.03, respectively. HOMA-IR is a surrogate measure of hepatic insulin sensitivity whereas SPISE index is a surrogate measure of whole-body insulin sensitivity (27, 28).

### Covariates: Cardiometabolic, Socioeconomic, and Lifestyle Factors

Heart rate and blood pressure were measured with semiautomated digital monitors at ages 15, 17, and 24 years as previously detailed (23, 25). A detailed assessment of fasting high-sensitivity C-reactive protein (hsCRP), low-density lipoprotein cholesterol (LDL-c), HDL-c, and triglycerides has been reported (coefficient of variation <5%) (23-25, 29) At the 17-year clinic visit, participants were briefly asked about their personal and family (mother, father, and siblings) medical history such as a history of hypertension, diabetes, high cholesterol, and vascular disease. All participants had attained puberty at the 17-year clinic visit using a time (years) to age at peak height velocity objective assessment derived from the Superimposition by Translation and Rotation mixed-effects growth curve analysis (25, 30).

Each participant’s mother’s socioeconomic status was grouped according to the 1991 British Office of Population and Census Statistics classification (31). Questionnaires to assess smoking behavior were administered at the 15-, 17-, and 24-year clinic visits. A specific question regarding whether participants smoked in the last 30 days was used as an indicator of current smoking status. Sedentary time, light physical activity, and moderate-to-vigorous physical activity were assessed with ActiGraph (LLC) accelerometer worn on the waist for 7 consecutive days at 15-year clinic visits whereas at 24 years movement behavior was assessed using an ActiGraph GT3X + accelerometer device worn for 4 consecutive days (32).

### Statistical Analysis

Cohort descriptive characteristics were summarized as means and SD, medians, and interquartile ranges, or frequencies and percentages. We explored sex differences using independent *t* tests, Mann-Whitney *U* tests, or chi-square tests for normally distributed, skewed, or dichotomous variables, respectively. Multicategory variables were analyzed using a one-way analysis of variance. Normality was assessed by histogram curve, quantile-quantile plot, and Kolmogorov-Smirnov tests with a *P* value less than .05. We conducted a logarithmic transformation of skewed variables and confirmed normality prior to further analysis.

#### Analyses of longitudinal associations

We examined the separate longitudinal associations of each of the 9-year cumulative total fat mass, trunk fat mass, lean mass, and BMI progression (ages 15-24 years) with the risk of each of hyperglycemia, hyperinsulinemia, and high insulin resistance at ages 15, 17, and 24 years using generalized linear mixed-effect models (GLMM) with logit link. The optimal model with the lowest Bayesian information criteria was one with sex as a main effect, a random intercept modeled for the participants to account for within-individual correlations. While the GLMM is robust for handling missing at random predictor and covariate data, we elected to additionally conduct 20 cycles of multiple imputations to account for missing data. The GLMM accounted for baseline body composition exposures, metabolic outcomes, and covariates and their repeated measures. For total fat mass, trunk fat mass, and lean mass variable analyses, model 1 was unadjusted. Model 2 was adjusted for sex and other time-varying covariates measured both at baseline and follow-up such as age, LDL-c, triglyceride, hsCRP, HDL-c, and heart rate, in addition to systolic blood pressure, glucose, insulin, fat mass, or lean mass, depending on the exposure or outcome. Model 3 was an additional adjustment for the lifestyle factors sedentary time, light physical activity, moderate-to-vigorous physical activity, smoking status, family history of hypertension/diabetes/high cholesterol/vascular disease, and socioeconomic status.

#### Cross-lagged temporal path analyses

We used structural equation modeling with an autoregressive cross-lagged design to examine the separate temporal associations of total fat mass or lean mass with insulin resistance (HOMA-IR). The cross-lagged models first tested the separate associations of total fat mass or lean mass at 15 years with insulin resistance at 17 years. Next, the associations of insulin resistance at 15 years with total fat mass or lean mass at 17 years were examined. Thereafter, we examined the separate associations of total fat mass or lean mass at 17 years with insulin resistance at 24 years. Last, the associations of insulin resistance at 17 years with total fat mass or lean mass at 24 years were examined. These models were adjusted for all the covariates measured at 15 and 17 years as listed earlier. In the cross-lagged design, the potential association could be total fat mass or lean mass leading to insulin resistance risks, insulin resistance risks leading to total fat mass or lean mass, or bidirectional associations of total fat mass or lean mass with insulin resistance risks. If a path from total fat mass or lean mass at time t-1 (15 years) to insulin resistance at time t-2 (17 years) reaches statistical significance (*P* < .05), changes in the earlier variables are considered to temporally precede changes in the latter, and vice versa. Likewise, if a path from total fat mass or lean mass at time t-2 (17 years) to insulin resistance at time t-3 (24 years) reaches statistical significance (*P* < .05), changes in the earlier variables are considered to temporally precede changes in the latter, and vice versa. A stronger predictive effect is determined by a larger standardized regression coefficient. Error terms were included in the cross-lagged model.

#### Mediation path longitudinal analyses

Last, mediating path analyses using structural equation models separately examined the mediating role of cumulative lipids, hsCRP, and fat mass or lean mass depending on the exposure on the longitudinal associations of cumulative fat mass and lean mass with insulin resistance from age 15 through 24 years. The mediation analysis was conducted in line with the Guideline for Reporting Mediation Analyses of Randomized Trials and Observational Studies (AGReMA) (33). Analyses were adjusted for age, sex, HDL-c, LDL-c, triglycerides, hsCRP, family history of hypertension and cardiovascular diseases, smoking status, heart rate, systolic blood pressure, sedentary time, light physical activity, moderate-to-vigorous physical activity , total fat mass, or lean mass depending on the exposure. The path models had 3 equations per regression analysis: the longitudinal associations of cumulative total fat mass or lean mass with cumulative lipids, blood pressure, or inflammation (equation 1); the longitudinal associations of cumulative lipids, blood pressure, or inflammation with insulin resistance (equation 2); and the longitudinal associations of cumulative total fat mass or lean mass with insulin resistance (equation 3, total effect), and equation 3′(direct effect) accounted for the mediating role of cumulative lipids, blood pressure, or inflammation on the longitudinal associations of cumulative total fat mass or lean mass with cumulative insulin resistance. The proportion of mediating or suppressing roles was estimated as the ratio of the difference between equation 3 and equation 3′ or the multiplication of equations 1 and 2 divided by equation 3 and expressed as a percentage. A mediating or indirect role is confirmed when there are statistically significant associations between (a) the predictor and mediator, (b) the predictor and outcome, (c) the mediator and outcome, and (d) the longitudinal association between the predictor and outcome variable was attenuated on inclusion of the mediator (34). However, when the magnitude of the longitudinal association between the predictor and outcome is increased on inclusion of a third variable, a suppression is confirmed (34). This means that suppression occurs when the mediational path has an opposite effect, that is, instead of a decrease in the point estimate of the direct effect between an exposure and an outcome in relation to the total effect, there is rather an increase in the direct effect above the total effect’s point estimate (34). We considered a statistically significant mediation or suppression of less than 1% as minimal, and 1% or greater as partial. Path analyses were conducted with 1000 bootstrapped samples (35, 36).

Collinearity diagnoses were performed and accepted results with a variance inflation factor less than 2, considered differences and associations with a 2-sided *P* value less than .05 as statistically significant, and drew conclusions based on effect estimates and their CI. Covariates were identified based on previous studies (13, 17, 24, 31, 37-40) We applied Sidak correction for potential multiple comparisons. Analyses involving 30% of a sample of 10 000 ALSPAC children at 0.8 statistical power, 0.05 α, and 2-sided *P* value would show a minimum detectable effect size of 0.053 SDs if they had relevant exposure for a normally distributed quantitative variable (41). All statistical analyses were performed using SPSS statistics software, version 27.0 (IBM Corp), and mediation analyses structural equation modeling was conducted using IBM AMOS version 27.0.

## Results

**Figure 1.**
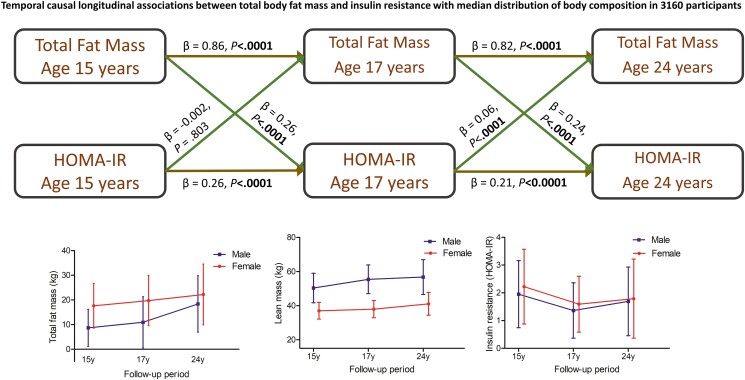
Trajectories of fat mass, lean mass, and insulin resistance (median and interquartile ranges) from ages 15 through 24 years and autoregressive cross-lagged temporal causal associations of fat mass with insulin resistance. The cross-lagged model was adjusted for sex, family history of hypertension/diabetes/high cholesterol/vascular disease, socioeconomic status, sedentary time, light physical activity, moderate to vigorous physical activity, and variables measured at ages 15 and 17 years such as age, low-density lipoprotein cholesterol, triglycerides, high-sensitivity C-reactive protein, high-density lipoprotein cholesterol, heart rate, smoking status, systolic blood pressure, and lean mass. Skewed variables were logarithmically transformed before analyses. A 2-sided *P* value less than .05 is considered statistically significant. β is the standardized regression coefficient. Autoregressive cross-lagged longitudinal analyses were conducted using structural equation temporal causal path models. HOMA-IR, homeostatic model assessment for insulin resistance.

Altogether, 3160 participants who had complete body composition and metabolic outcomes at age 15 years were included. From age 15 through 24 years, fat mass and lean mass increased linearly both in male and female participants (Table 1 and Fig. 1). Fasting glucose, insulin, and HOMA-IR had a U-shaped increase from age 15 through 24 years with the nadir at age 17 years (see Table 1 and Fig. 1). The prevalence of obesity increased 5-fold both in males and females during growth from age 15 to 24 years. Other characteristics are described in Table 1.

**Table 1.** Descriptive characteristics of 3160 participants who had complete body composition and insulin resistance at age 15 years clinic visits

Age at clinic visits/follow-up	15 y			17 y			24 y		
Variables	Male (n = 1546)	Female (n = 1614)	*P*	Male (n = 1167)	Female (n = 1350)	*P*	Male (n = 787)	Female (n = 1093)	*P*
***Anthropometry***									
Age at clinic visit, y, mean (SD)	15.41 (0.26)	15.43 (0.28)	.077	17.74 (0.38)	17.74 (0.37)	.876	24.56 (0.78)	24.44 (0.75)	.001
Height, m, mean (SD)	1.75 (0.08)	1.65 (0.06)	<.001	1.79 (0.07)	1.66 (0.06)	<.0001	1.80 (0.07)	1.66 (0.06)	<.0001
*^a^* Weight, kg	62.80 (13.5)	58.0 (12.3)	<.0001	70.60 (14.75)	61.00 (13.68)	<.0001	79 (17.67)	65.30 (17.30)	<.0001
Attained puberty (n, %)	1400 (95.4)	1519 (>99.9)	<.001	NA			NA		
***Body composition***									
*^a^* Total fat mass, kg	8.67 (7.51)	17.59 (9.07)	<.0001	10.91 (10.41)	19.71 (10.19)	<.0001	18.35 (11.43)	22.18 (12.32)	<.0001
*^a^* Trunk fat mass, kg	3.79 (3.69)	8.05 (5.12)	<.0001	5.63 (5.68)	9.68 (5.67)	<.0001	9.09 (6.78)	10.06 (6.92)	<.001
*^a^* Lean mass, kg	50.34 (8.64)	36.99 (4.90)	<.0001	55.39 (8.43)	37.96 (5.08)	<.0001	56.74 (10.22)	41.05 (6.71)	<.0001
*^a^* BMI, kg/m2	20.42 (3.45)	21.23 (3.97)	<.001	21.71 (4.09)	22.18 (4.05)	.004	24.22 (4.95)	23.65 (5.95)	.146
Overweight BMI 25-29.9	136 (8.8)	200 (12.4)	<.001	171 (14.9)	212 (16.1)	.006	248 (31.6)	233 (21.6)	.758
Obese BMI >29.9	36 (2.3)	59 (3.7)	<.001	56 (4.9)	97 (7.4)	.006	81 (10.3)	171 (15.9)	.758
***Fasting plasma metabolic indices***									
High-density lipoprotein, mmol/L, mean (SD)	1.22 (0.27)	1.36 (0.30)	<.001	1.19 (0.25)	1.35 (0.31)	<.001	1.40 (0.36)	1.66 (0.42)	<.001
Low-density lipoprotein, mmol/L, mean (SD)	1.99 (0.52)	2.18 (0.57)	<.001	1.99 (0.56)	2.21 (0.64)	<.001	2.47 (0.76)	2.43 (0.76)	.333
*^a^* Triglycerides, mmol/L	0.72 (0.38)	0.76 (0.37)	<.001	0.74 (0.37)	0.75 (0.38)	.502	0.88 (0.55)	0.80 (0.42)	<.001
Glucose, mmol/L, mean (SD)	5.30 (0.40)	5.13 (0.36)	<.001	5.13 (0.40)	4.90 (0.37)	<.001	5.47 (0.79)	5.21 (0.56)	<.001
Hyperglycemia (>6.1 mmol/L) (n, %)	18 (1.2)	11 (0.7)	.191	12 (1.2)	6 (0.6)	.158	58 (7.8)	36 (3.8)	<.001
*^a^* Insulin, mU/L	8.18 (4.88)	9.74 (5.66)	<.0001	5.95 (3.97)	7.24 (4.32)	<.0001	7.11 (4.94)	7.84 (5.67)	<.001
Hyperinsulinemia (>11.78mU/L) (n, %)	304 (19.7)	516 (32.0)	<.001	115 (11.6)	152 (14.9)	.030	133 (17.9)	206 (21.8)	.050
*^a^* Insulin resistance (HOMA-IR)	1.95 (1.21)	2.22 (1.35)	<.0001	1.36 (1.00)	1.59 (1.01)	<.001	1.69 (1.24)	1.79 (1.43)	.119
Insulin sensitivity (SPISE)	9.31 (2.10)	8.92 (2.06)	<.001	8.49 (1.97)	8.51 (2.10)	.859	7.44 (1.97)	7.95 (2.17)	<.001
*^a^* High-sensitivity C-reactive protein, mg/L	0.38 (0.67)	0.39 (0.67)	.574	0.48 (0.79)	0.65 (1.40)	<.001	0.65 (1.19)	0.98 (1.99)	<.001
***Vascular measures***									
Heart rate, beat/min, mean (SD)	71 (12)	77 (12)	<.001	63 (9)	67 (10)	<.001	65 (10)	68 (10)	<.001
Systolic blood pressure, mm Hg, mean (SD)	127 (10)	121 (11)	<.001	120 (9)	110 (8)	<.001	123 (10)	112 (9)	<.001
Diastolic blood pressure, mm Hg, mean (SD)	68 (9)	66 (8)	<.001	63 (6)	65 (6)	<.001	68 (8)	66 (8)	<.001
***Lifestyle and sociodemographic factors***									
Smoking status (n, %)	208 (13.9)	318 (20)	<.001	251 (25.1)	338 (29.2)	.033	226 (29.2)	301 (27.7)	.498
Family history of H-D-C-V (n,%)	326 (29.2)	386 (29.8)	.788	NA			NA		
Sedentary time (min/d), mean (SD)	353 (74)	361 (70)	.009	461 (93)	482 (82)	<.001	529 (79)	520 (85)	.250
Light physical activity (min/d), mean (SD)	368 (60)	369 (58)	.618	290 (67)	272 (64)	<.001	142 (56)	149 (53)	.163
MVPA (min/d), mean (SD)	67 (33)	48 (23)	<.001	56 (30)	41 (30)	<.001	56 (35)	49 (28)	.030
Ethnicity White (n, %)	1359 (95.8)	1409 (96.2)	.356	NA			NA		
Maternal social economic status (n, %)			.077	NA			NA		
*Professional*	59 (8.1)	28 (4.0)							
*Managerial and technical*	293 (40)	266 (38.2)							
*Skilled nonmanual*	239 (32.7)	261 (37.5)							
*Skilled manual*	13 (1.8)	18 (2.6)							
*Partly skilled*	105 (14.3)	98 (14.1)							
*Unskilled*	23 (3.1)	25 (3.6)							

Values are means (SD) and *^a^*median (interquartile range) except for lifestyle factors and ethnicity. Differences between sexes were tested using the *t* test for normally distributed continuous variables, Mann-Whitney *U* test for skewed continuous variables, chi-square test for dichotomous variable, and analysis of covariance for multicategory variable. A 2-sided *P* value less than .05 is considered statistically significant. HOMA-IR was computed from (fasting insulin × fasting glucose/22.5); SPISE was computed from [600×HDL-c^0.185^/(triglycerides^0.2×^BMI^1.338^)]. *P* value for sex differences.

Abbreviations: BMI, body mass index; H-D-C-V, hypertension/diabetes/high cholesterol/vascular disease; HDL-c, high-density lipoprotein cholesterol; HOMA-IR, homeostatic model assessment of insulin resistance; MVPA, moderate-to-vigorous physical activity; NA, not available/applicable; SPISE, single-point insulin sensitivity estimator.

^*a*^Median (interquartile range).

### Longitudinal Associations of Body Composition With Risk of Metabolic Alteration

After full adjustments for lifestyle and cardiometabolic factors, cumulative total fat mass (odds ratio [OR] 1.12 [95% CI, 1.11-1.13]), trunk fat mass (1.21 [1.19-1.23]), and BMI (1.26 [1.23-1.27]) from ages 15 through 24 years were associated with a progressively worsening risk of high insulin resistance, as well as hyperglycemia and hyperinsulinemia (Table 2). Cumulative increase in lean mass (0.98 [0.98-0.99]) was associated with a lower risk of high insulin resistance, as well as hyperinsulinemia but there was no statistically significant association with hyperglycemia (see Table 2).

**Table 2.** Longitudinal associations of cumulative body composition with risk of progressive hyperglycemia, hyperinsulinemia, and elevated insulin resistance from ages 15 through 24 years among 3160 participants

N = 3160	Hyperglycemia (>6.1 mmol/L)		Hyperinsulinemia (>11.78 mU/L)		Elevated insulin resistance	
	OR (95% CI)	*P*	OR (95% CI)	*P*	OR (95% CI)	*P*
**Continuous cumulative predictor variables from ages 15-24 y**						
**Total fat mass, kg**						
Model 1	1.06 (1.05-1.07)	<.**0001**	1.08 (1.07-1.09)	<.**0001**	1.10 (1.09-1.11)	<.**0001**
Model 2	1.04 (1.03-1.05)	<.**0001**	1.08 (1.07-1.09)	<.**0001**	1.12 (1.11-1.13)	<.**0001**
Model 3	1.04 (1.03-1.05)	<.**0001**	1.09 (1.08-1.09)	<.**0001**	1.12 (1.11-1.13)	<.**0001**
**Trunk fat mass, kg**						
Model 1	1.10 (1.09-1.12)	<.**0001**	1.15 (1.14-1.17)	<.**0001**	1.19 (1.17-1.21)	<.**0001**
Model 2	1.08 (1.07-1.10)	<.**0001**	1.14 (1.12-1.16)	<.**0001**	1.21 (1.19-1.23)	<.**0001**
Model 3	1.07 (1.06-1.09)	<.**0001**	1.13 (1.13-1.16)	<.**0001**	1.21 (1.19-1.23)	<.**0001**
**Lean mass, kg**						
Model 1	1.01 (0.99-1.02)	.190	0.99 (0.99-1.01)	.504	1.00 (0.99-1.01)	.461
Model 2	1.01 (0.99-1.01)	.494	0.98 (0.98-0.99)	.**004**	0.98 (0.98-0.99)	.**010**
Model 3	1.01 (0.99-1.02)	.238	0.98 (0.98-0.99)	.**007**	0.98 (0.98-0.99)	.**009**
**BMI, kg/m2**						
Model 1	1.08 (1.06-1.11)	<.**001**	1.17 (1.14-1.19)	<.**0001**	1.21 (1.19-1.23)	<.**0001**
Model 2	1.07 (1.05-1.09)	<.**0001**	1.18 (1.15-1.20)	<.**0001**	1.27 (1.24-1.29)	<.**0001**
Model 3	1.07 (1.05-1.09)	<.**001**	1.19 (1.16-1.21)	<.**0001**	1.26 (1.23-1.27)	<.**0001**

For continuous variable analyses, model 1 was unadjusted. Model 2 was adjusted for sex, and other time-varying covariates measured both at baseline and follow-up such as age, low-density lipoprotein cholesterol, triglycerides, high-sensitivity C-reactive protein, high-density lipoprotein cholesterol, heart rate, systolic blood pressure, in addition to glucose, insulin, fat mass, or lean mass depending on the exposure or outcome. Model 3 was an additional adjustment for lifestyle factors sedentary time, light physical activity, moderate to vigorous physical activity, smoking status, family history of hypertension/diabetes/high cholesterol/vascular disease, and socioeconomic status. ORs were computed from the generalized linear mixed-effect model with logit link for repeated measures. A 2-sided *P* value less than .05 is considered statistically significant and are bolded. Multiple testing was corrected with Sidak correction. Multiple imputations were used to account for missing variables. The BMI predictor model was not adjusted for lean mass and fat mass; the insulin resistance outcome model was not adjusted for insulin and glucose. HOMA-IR was computed from (fasting insulin × fasting glucose/22.5). Elevated insulin resistance describes greater than or equal to the 75th percentile.

Abbreviations: BMI, body mass index; HOMA-IR, homeostatic model assessment of insulin resistance; OR, odds ratio.

Among males and females, increased total fat mass, trunk fat mass, and BMI during growth from age 15 to 24 years were associated with increased fasting insulin concentration and insulin resistance but with decreased fasting glucose (Supplementary Table S1) (42). Increased lean mass was associated with increased fasting insulin and insulin resistance in females but not in males (see Supplementary Table S1) (42).

Among normal-weight and participants with overweight/obesity, increased total fat mass and trunk fat mass during growth from age 15 to 24 years were associated with increased fasting insulin concentration and insulin resistance but with decreased fasting glucose (Supplementary Table S2) (42). Increased lean mass was associated with increased fasting insulin and insulin resistance among participants who were overweight/obese but with decreased insulin resistance in normal-weight participants (see Supplementary Table S2) (42).

Cumulatively increased total fat mass was inversely associated with SPISE index from age 15 to 24 years (unstandardized regression coefficient −8.26 [95% CI, −8.37 to −8.16]) (*P* < .0001). Cumulatively increased trunk fat mass was inversely associated with SPISE index from age 15 to 24 years (−7.43 [−7.52 to −7.34]) (*P* < .0001). Cumulatively increased BMI was inversely associated with SPISE index from age 15 to 24 years (−28.76 [−30.96 to −26.57]) (*P* < .0001). Cumulatively increased lean mass was inversely associated with SPISE index from age 15 to 24 years (−2.65 [−3.02 to −2.28] (*P* < .0001).

### Temporal Path Associations of Fat Mass or Lean Mass With Insulin Resistance

Total fat mass, lean mass, and insulin resistance (HOMA-IR) at age 15 years were directly associated with their individual variables at age 17 years (Table 3 and Fig. 1). Moreover, total fat mass, lean mass, and insulin resistance at age 17 years were directly associated with their respective variables at age 24 years (see Table 3 and Fig. 1).

**Table 3.** Autoregressive cross-lagged temporal causal longitudinal analyses of fat mass, lean mass, and insulin resistance in relation to insulin resistance at ages 15, 17, and 24 years

3160 Participants				
Insulin resistance				
Autoregressive	B	β	SE	*P*
Total FM T1⇒Total FM T2	0.834	.863	0.009	**<**.**0001**
Total FM T2 ⇒ Total FM T3	0.543	.827	0.008	**<**.**0001**
Lean mass T1 ⇒ Lean mass T2	0.744	.667	0.008	**<**.**0001**
Lean mass T2 ⇒ Lean mass T3	0.921	.203	0.010	**<**.**0001**
HOMA-IR T1 ⇒ HOMA-IR T2	0.251	.258	0.016	**<**.**0001**
HOMA-IR T2 ⇒ HOMA-IR T3	0.289	.213	0.034	**<**.**0001**
***Cross-lagged***				
Total FM T1 ⇒ HOMA-IR T2	0.209	.261	0.017	**<**.**0001**
HOMA-IR T1 ⇒ Total FM T2	−0.002	−.002	−0.250	.803
Total FM T2 ⇒ HOMA-IR T3	0.266	.237	8.648	**<**.**0001**
HOMA-IR T2 ⇒ Total FM T3	0.050	.064	0.009	**<**.**0001**
Lean mass T1 ⇒ HOMA-IR T2	−0.141	−.055	0.064	**<**.**028**
HOMA-IR T1 ⇒ Lean mass T2	0.002	.004	0.002	.421
Lean mass T2 ⇒ HOMA-IR T3	0.251	.081	0.091	.**006**
HOMA-IR T2 ⇒ Lean mass T3	0.009	.022	0.004	.**020**

Model was adjusted for baseline age, sex, low-density lipoprotein cholesterol, triglycerides, high-sensitivity C-reactive protein, high-density lipoprotein cholesterol, heart rate, smoking status, systolic blood pressure, family history of hypertension/diabetes/high cholesterol/vascular disease, socioeconomic status, sedentary time, light physical activity, moderate to vigorous physical activity, in addition to fat mass or lean mass depending on predictor. Skewed variables were logarithmically transformed before analyses. A 2-sided *P* value less than .05 is considered statistically significant and are bolded. Autoregressive cross-lagged longitudinal analyses were conducted using structural equation temporal causal path models.

Abbreviations: Time T1, aged 15 years; Time T2, aged 17 years; Time T3, aged 24 years. B, unstandardized regression; β, standardized regression; FM, fat mass; HOMA-IR, homeostatic model assessment of insulin resistance.

Higher total fat mass at 15 years was associated with higher insulin resistance at 17 years, but higher insulin resistance at 15 years was not associated with higher total fat mass at 17 years (see Table 3 and Fig. 1). Higher total fat mass at 17 years was bidirectionally associated with higher insulin resistance at 24 years (see Table 3 and Fig. 1). Higher lean mass at 15 years was associated with lower insulin resistance at 17 years, but higher insulin resistance at 15 years was not associated with higher lean mass at 17 years (see Table 3 and Fig. 1). Higher lean mass at 17 years was bidirectionally associated with higher insulin resistance at 24 years (see Table 3 and Fig. 1).

### Mediating or Suppressing Effects of Lipids, Systolic Blood Pressure, and Inflammation in the Longitudinal Associations of Total Fat Mass and Lean Mass with Insulin Resistance

**Figure 2.**
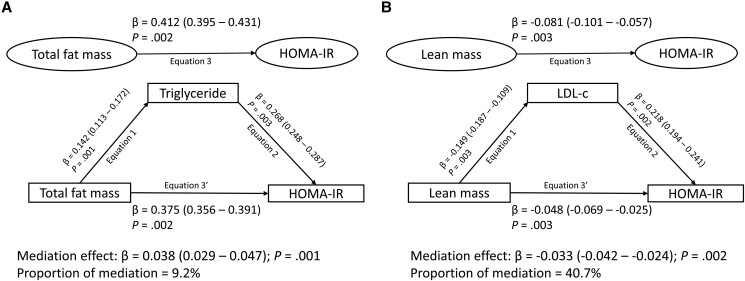
Longitudinal mediating effect of A, triglycerides and B, low-density lipoprotein cholesterol in the associations of fat mass or lean mass with insulin resistance from ages 15 through 24 years. Mediation structural equation model was adjusted for sex, family history of hypertension/diabetes/high cholesterol/vascular disease, socioeconomic status, and time-varying covariates measured both at baseline and follow-up such as age, heart rate, systolic blood pressure, smoking status, high-density lipoprotein cholesterol, high-sensitivity C-reactive protein, sedentary time, light physical activity, and moderate-to-vigorous physical activity, with additional adjustments for fat mass, lean mass, low-density lipoprotein cholesterol, or triglycerides depending on the predictor or mediator. β is the standardized regression coefficient. *P* values less than .05 were considered statistically significant. When the magnitude of the longitudinal association between the predictor and outcome is decreased on inclusion of a third variable, a mediation is confirmed. HOMA-IR, homeostatic model assessment for insulin resistance; LDL-c, low-density lipoprotein cholesterol.

Cumulative HDL-c, LDL-c, triglycerides, systolic blood pressure, and lean mass partially mediated (1.3%-9.2% mediation) the longitudinal associations of increased fat mass with increased insulin resistance (Table 4 and Fig. 2) after full adjustments for covariates. There was no statistically significant mediating effect of hsCRP on the relationship of fat mass with insulin resistance.

**Table 4.** Mediating or suppressing role of cumulative lipids, inflammation, and systolic blood pressure on the longitudinal associations of total fat mass and lean mass with and insulin resistance progression from ages 15 through 24 years for 3160 participants

	Cumulative insulin resistance ages 15-24 y						
	Total effect		Direct effect		Indirect effect		Mediation or suppression, %
*Mediators*	β (95% CI)	*P*	β (95% CI)	*P*	β (95% CI)	*P*	
**Cumulative total fat mass**							
HDL-c	.481 (0.465 to 0.498)	.002	.462 (0.446 to 0.478)	.002	.019 (0.015 to 0.023)	.**001**	**4.0 mediation**
LDL-c	.442 (0.425 to 0.459)	.002	.429 (0.410 to 0.447)	.002	.013 (0.009 to 0.018)	.**001**	**2.9 mediation**
Triglycerides	.412 (0.395 to 0.431)	.002	.375 (0.356 to 0.391)	.002	.038 (0.029 to 0.047)	.**001**	**9.2 mediation**
High-sensitivity CRP	.456 (0.440 to 0.473)	.001	.463 (0.445 to 0.482)	.002	−.007 (−0.015 to 0.000)	.067	1.5
Systolic blood pressure	.368 (0.353 to 0.387)	.001	.357 (0.342 to 0.375)	.001	.011 (0.007 to 0.015)	.**002**	**3.0 mediation**
Lean mass	.468 (0.448 to 0.489)	.001	.462 (0.444 to 0.480)	.001	.006 (0.002 to 0.010)	.**004**	**1.3 mediation**
**Cumulative lean mass**							
HDL-c	−.073 (−0.095 to −0.050)	.002	−.111 (−0.132 to −0.087)	.002	.038 (0.030 to 0.047)	.**001**	**52.1 suppression**
LDL-c	−.081 (−0.101 to −0.057)	.003	−.048 (−0.069 to −0.025)	.003	−.033 (−0.042 to −0.024)	.**002**	**40.7 mediation**
Triglycerides	−.081 (−0.105 to −0.055)	.002	−.089 (−0.109 to −0.068)	.002	.008 (−0.010 to 0.021)	.349	10
High-sensitivity CRP	−.075 (−0.096 to −0.051)	.002	−.067 (−0.088 to −0.041)	.003	−.008 (−0.015 to −0.003)	.**008**	**10.6 mediation**
Systolic blood pressure	.009 (−0.011 to 0.031)	.413	−.076 (−0.096 to −0.056)	.002	.085 (0.078 to 0.092)	.002	944.4
Fat mass	.197 (0.172 to 0.226)	.001	.030 (0.009 to 0.051)	.005	.163 (0.152 to 0.184)	.**002**	**84.5 mediation**

Mediation structural equation model was adjusted for sex, family history of hypertension/diabetes/high cholesterol/vascular disease, socioeconomic status, and time-varying covariates measured both at baseline and follow-up such as age, heart rate, smoking status, light physical activity, and moderate-to-vigorous physical activity, with additional adjustments for fat mass, lean mass, insulin resistance, high sensitivity CRP, HDL cholesterol, LDL cholesterol, or triglycerides depending on the mediator. *P* values less than .05 were considered statistically significant and are bolded. When the magnitude of the longitudinal association between the predictor and outcome is increased on inclusion of a third variable, a suppression is confirmed; however, when decreased it is mediation.

Abbreviations: β, standardized regression coefficient; CRP, C-reactive protein; HDL, high-density lipoprotein cholesterol; LDL, low-density lipoprotein cholesterol.

With a mediating effect of 41%, LDL-c partially mediated the associations of cumulatively increased lean mass with decreased insulin resistance (see Table 4 and Fig. 2). Cumulative increased fat mass strongly mediated (85% mediation) the longitudinal associations of increased lean mass with increased insulin resistance (see Table 4).

## Discussion

In the largest and longest follow-up study of adolescents with objectively measured body composition and repeated fasting blood samples from mid-adolescence to young adulthood, the following were observed. First, increased total body fat mass and trunk fat mass were separately associated with an increased risk of hyperinsulinemia and insulin resistance. Second, higher total fat mass in mid-adolescence may temporally precede higher insulin resistance by late adolescence, progressing to a bidirectional relationship between total fat mass and insulin resistance by young adulthood. Third, increasing lipids partially mediated the association between fat mass and insulin resistance. Last, increased lean mass may protect against increased insulin resistance and hyperinsulinemia.

### Fat Mass and Metabolic Alterations

More than a quarter of a billion children and adolescents might be living with obesity by 2030 as estimated by the World Obesity Federation (4). Previous evidence on the causal link between obesity and insulin resistance in the pediatric population has relied on surrogate measures of obesity such as BMI and waist circumference, but these measures poorly discriminate between fat mass and lean mass (3, 6-10). A direct measure of fat mass adiposity using DXA has been limited to cross-sectional studies in small sample-sized populations and a few short-term longitudinal studies (8, 10, 11). Moreover, metabolic alteration such as glucose intolerance has been recorded in children and adolescents who are overweight or obese but evidence in a normal-weight young population is conflicting (5, 9, 11, 12). Thus, large-scale, long-term longitudinal studies of directly measured fat mass in relation to metabolic indices are warranted to clarify the independent role of fat mass in metabolic alteration, especially in the normal-weight pediatric population (2, 3, 9, 10).

In 564 primary school children from Canada aged 8 to 10 years and followed up for 2 years, who had at least 1 parent with BMI greater than 30 kg/m2, every additional 1% of body fat measured with DXA at baseline was associated with a 3.2% increase in insulin resistance (HOMA-IR) (12). In this present study with a 6 times larger cohort and longer follow-up of 9 years, we observed that both increased total fat mass and trunk fat mass were longitudinally associated with the risk of worsening hyperinsulinemia and high insulin resistance during growth from ages 15 to 24 years. This result was consistent both in normal-weight participants as well as those who are overweight or obese, suggesting that fat mass at physiological concentration may be a strong risk factor for the development of insulin resistance independent of physical activity. We observed that trunk fat mass doubled the risk of high insulin resistance when compared to total fat mass, buttressing the finding that truncal adiposity may be more metabolically deleterious (9). Nearly all participants had attained puberty at baseline age 15 years, and controlling for puberty did not alter the results (data not shown) (17).

Several experimental studies have postulated pathways for the relationship between obesity and insulin resistance such as increased inflammation, dysfunctional adipose tissue, hormones, hypothalamus-pituitary-adrenal-fat axis abnormalities, sympathetic nervous system overdrive, decreased brown or beige adipocytes, lipotoxicity or lipoapoptosis, mitochondrial dysfunction, and endoplasmic reticulum stress (9, 10, 15). Many of these mechanistic explanations are from animal models, necessitating new pathways in future research, especially in human studies (10, 15). In the present study, we observed that increased lipids, especially triglycerides, explained 9% of the relationship between fat mass and insulin resistance in the cohort of largely normal-weight participants. Inflammation assessed with hsCRP did not mediate the relationship between fat mass and insulin resistance, especially after accounting for physical activity, nonetheless, further studies with other inflammatory markers are warranted (10, 40, 43, 44). Although the prevalence of smoking doubled during growth from mid-adolescence to young adulthood, it only confounded the relationship between cumulative fat mass and insulin resistance by approximately 4% (data not shown). We observed that higher total fat mass in mid-adolescence temporally preceded higher insulin resistance by late adolescence; however, higher total fat mass in late adolescence was bidirectionally associated with higher insulin resistance in young adulthood. These findings suggest that mid-adolescence might be an important time to interrupt the vicious cascade of higher fat mass and insulin resistance, but further experimental studies are needed (5). A recent large-scale longitudinal study in more than 6000 children followed up until young adulthood concluded that engaging in at least 3 to 4 hours/day of light-intensity physical activity may decrease body fat mass by a maximum of 15% (45). This decrease may be clinically relevant in lowering insulin resistance and improving insulin sensitivity in the young population (5, 46).

### Lean Mass and Metabolic Alterations

In a cross-sectional study of US adults aged 41 years, skeletal muscle mass estimated by bioelectrical impedance was associated with lower insulin resistance (47). A recent review summarized that the relationship between exercised-improved glucose homeostasis and increased skeletal muscle mass may be concurrent but not necessarily causally associated (48). In this study, we observed that increased lean mass was associated with a 2% reduced risk of hyperinsulinemia and insulin resistance in the whole cohort. This was confirmed in normal-weight participants, among whom we observed that increased lean mass was associated with lower insulin resistance. Furthermore, the mediating path analyses suggest that the association of increased lean mass and insulin resistance, especially in participants who are overweight, may be explained by the residual effect of fat mass (85% mediation). We observed that higher lean mass in mid-adolescence temporally preceded lower insulin resistance by late adolescence; however, higher lean mass in late adolescence was bidirectionally associated with higher insulin resistance in young adulthood possibly because of the significant increase in fat mass between late adolescence and young adulthood. From age 15 to 17 years, an acute physiologic response to postpubertal changes (17) was observed by which lean mass potentially reduced insulin resistance and had a carry-over cumulative effect, although the relationship between lean mass and insulin resistance from ages 17 to 24 years was positive rather than negative. In a recent study, we observed that increased physical activity was paradoxically associated with reduced HDL-c and that physical activity was associated with increased lean mass, reduced fat mass, and decreased insulin resistance (49). However, the 52% suppressive effect of HDL-c on the association between lean mass and insulin resistance may relate to liver metabolism (50). Since HOMA-IR reflects hepatic insulin resistance and excessively elevated HDL-c has been associated with liver damage, it is likely that the increased lean mass effect on reducing insulin resistance is counteracted significantly by increased HDL-c after accounting for the role of physical activity (50). Increased levels of intramyocellular lipids content result in an accumulation of intracellular fatty acyl CoAs or other fatty acid metabolites that modulate local glucose metabolism and result in elevated insulin resistance of skeletal muscles (50, 51). Overall, increased lean mass from mid-adolescence might protect against worsening insulin resistance in the young population, and thus aerobic and resistance exercise interventions to increase muscle mass and decrease fat mass are warranted (2, 5, 10, 45, 48, 52).

### Strength and Limitations

The ALSPAC data set provides an extensive array of gold-standard and repeated measures of body composition and covariates throughout the follow-up period in a large pediatric population. Using advanced statistical models, we examined the potential temporal and causal explanatory pathway and consistency of the longitudinal findings for the first time in a large pediatric population. On the other hand, the present study had some limitations. We did not measure insulin sensitivity and secretion using gold-standard methods such as the clamp test (53), given that the feasibility of these measures in large epidemiologic studies is limited; nonetheless, we used a surrogate whole-body insulin sensitivity measure (SPISE) previously validated in the young population (28). The computation of the SPISE index includes the BMI variable, and BMI assesses both fat mass and lean mass, hence the SPISE index is highly correlated (−0.75 to −0.80) with total fat mass and trunk fat mass. Therefore SPISE index results should be cautiously interpreted; for example, an increased lean mass was associated with decreased whole-body insulin sensitivity (SPISE index) but associated with increased hepatic insulin sensitivity (HOMA-IR). Our participants were mostly White; therefore, we are unable to generalize our findings to other racial and ethnic groups. Moreover, as with all observational studies, residual biases due to unmeasured confounders may distort observed associations such as the unavailability of dietary records and energy intake.

## Conclusion

In a 9-year follow-up temporal and mediation study of several thousand adolescents, we observed that progressive increase in fat mass temporally preceded insulin resistance and was associated with a worsening risk of hyperinsulinemia and insulin resistance both in males and females as well as in normal-weight participants and those overweight and obese. An increase in triglycerides partially explains the relationship between increased fat mass and insulin resistance. Increased trunk fat mass doubled the risk of worsening insulin resistance when compared with total fat mass. Increased lean mass was protective of insulin resistance, especially in normal-weight participants, and thus may be targeted in future interventions. Mid-adolescence through late adolescence might be a crucial time for interrupting the vicious cascade of higher fat mass and insulin resistance by young adulthood.

## Data Availability

The informed consent obtained from ALSPAC participants does not allow the data to be made freely available through any third-party–maintained public repository. However, data used for this submission can be made available on request to the ALSPAC Executive. The ALSPAC data management plan describes in detail the policy regarding data sharing, which is through a system of managed open access. Full instructions for applying for data access can be found at http://www.bristol.ac.uk/alspac/researchers/access/. The ALSPAC study website contains details of all the data that are available (http://www.bristol.ac.uk/alspac/researchers/our-data/).

## Abbreviations

ALSPAC: Avon Longitudinal Study of Parents and Children
BMI: body mass index
DXA: dual-energy x-ray absorptiometry
GLMM: generalized linear mixed-effect models
H-D-C-V: hypertension/diabetes/high cholesterol/vascular disease
HDL-c: high-density lipoprotein cholesterol
HOMA-IR: homeostatic model assessment of insulin resistance
hsCRP: high-sensitivity C-reactive protein
LDL-c: low-density lipoprotein cholesterol
MVPA: moderate-to-vigorous physical activity
SPISE: single-point insulin sensitivity estimator
